# Use of Model-Based Compartmental Analysis and Theoretical Data to Further Explore Choice of Sampling Time for Assessing Vitamin A Status in Groups and Individual Human Subjects by the Retinol Isotope Dilution Method

**DOI:** 10.1093/jn/nxab061

**Published:** 2021-04-08

**Authors:** Michael H Green, Joanne Balmer Green

**Affiliations:** Department of Nutritional Sciences, College of Health and Human Development, The Pennsylvania State University, University Park, PA, USA; Department of Nutritional Sciences, College of Health and Human Development, The Pennsylvania State University, University Park, PA, USA

**Keywords:** mathematical modeling, retinol isotope dilution, vitamin A status, vitamin A total body stores, WinSAAM

## Abstract

**Background:**

An optimal blood sampling time for application of the retinol isotope dilution (RID) method for predicting vitamin A total body stores (TBS) (i.e., vitamin A status) has not been established.

**Objectives:**

Objectives were to identify sampling times that provide accurate estimates of TBS by RID in groups and individuals by applying compartmental modeling to data for theoretical adults and children.

**Methods:**

We selected previously generated hypothetical adults and children (20 per group) that had a wide range of assigned values for TBS and vitamin A kinetic parameters. We used the Simulation, Analysis and Modeling software to simulate individual kinetic responses; then we calculated geometric mean values for the RID equation coefficients and each individual's plasma retinol specific activity at various times, using those values to predict group mean and individual subject TBS. Predicted values for TBS were compared with assigned values.

**Results:**

Accurate estimates of group mean TBS were obtained at all sampling times from 1 to 30 d in both adults and children. For individuals, correlations between RID-predicted TBS and assigned values increased with time in the adults (*R*^2 ^= 0.80 at day 14, 0.96 at day 21, and 0.99 at day 28); a similar trend was observed for the children, with *R*^2 ^= 0.82 at day 7 and increasing to 0.97 at days 21 and 28 (*P* < 0.001 for all comparisons).

**Conclusions:**

Although no single, unique time provided the most accurate prediction of TBS for all individuals within these groups, applying the RID method at 21 or 28 d yielded predictions that were within 25% of assigned values for 90% or 95% of adults, respectively; corresponding values for children were 80% from 10 to 20 d, and 85% at 21 and 28 d. For most subjects, early times (<14 d for adults and <10 d for children) provided less accurate predictions.

See corresponding commentary on page 1680.

## Introduction

The retinol isotope dilution (RID) method, originally developed by Bausch and Rietz (1) and Furr et al. (2), is considered the best available indirect method for assessing vitamin A status [defined as either total liver reserves or total body stores (TBS)] in humans (3, 4); as currently used, it is generally assumed to be most reliable for estimating TBS in groups but the technique is also applied to predict stores in individuals. In recent work, the accuracy of RID predictions has been evaluated using theoretical subjects (5), and the method's underlying assumptions have been discussed in detail (6); in addition, the method continues to be applied in community settings to assess vitamin A status (7–10).

In spite of the usefulness of the RID method, there is as yet no consensus on the best time to apply the technique in children or adults; in fact, the time (or suggested time) between ingestion of a dose of stable isotope-labeled vitamin A and blood sampling for RID ranges from 3 d (11–14) to 21 d (2). Choice of sampling time is complicated by the fact that the longer the interval between tracer dosing and blood sampling, the greater are potential challenges with subject retention, intercurrent infection, and quantification of tracer. Also, if sampling is done later in subjects who have low stores and a high system fractional catabolic rate for vitamin A, TBS can be substantially overestimated (15).

In both early (2) and more recently developed RID equations for predicting TBS (16, 17), plasma retinol specific activity (SA_p_), the measured variable, is adjusted based on several factors that are included as coefficients in the equation (see Methods). Values for the coefficients have either been estimated from the literature (2, 12, 17, 18) or determined by compartmental modeling and the “super-subject” approach (5, 7, 9, 10). When that design is used to reduce sampling burden on individual subjects, modeling is applied to a composite (geometric mean) plasma retinol tracer dataset; this provides an estimate of TBS for the group and values for the RID coefficients that can be used with each subject's data for SA_p_ at a specific time to predict TBS in individuals. By applying the super-subject method to data for theoretical children who had a wide range of values for TBS and vitamin A kinetic parameters, we showed in Ford et al. (5) that this approach provided predictions that were within 25% of the assigned (known) TBS for 94% of the subjects at 7 d and for 78% at 4 d.

Here, we applied model-based compartmental analysis to simulated data for theoretical adults and children that had a wide range of vitamin A kinetics and TBS to further explore the identification of an optimal sampling time(s) for predicting TBS by RID. As in other studies (19–22) we used theoretical subjects based on published vitamin A kinetics and TBS so that we could evaluate the accuracy of our results, something that cannot be done in human studies. Our results indicate that accurate estimates of group mean TBS were obtained at any sampling time, whereas for individuals, the most accurate results for most subjects were obtained when the RID equation was applied ≥14 d after dosing; however, at least in these 2 groups of theoretical subjects, there was no single, unique time that provided the most accurate prediction of TBS for all individuals. Beyond these findings, our analyses provide estimates for the RID equation coefficients that could be useful to researchers when it is not feasible to determine such values for their specific study group.

## Methods

### Theoretical subjects

We selected 40 previously studied theoretical subjects (20 adults and 20 children) who had a wide range of assigned values for TBS (<50 to >2000 μmol) and variable vitamin A kinetics. Eight of the adults were selected from Ford et al. (21) and 12 from Green et al. (22); 4 of the children were first described in Ford et al. (21) and 16 were from reference 5, with kinetic parameters for the latter subjects adjusted to reflect results obtained in reference 9. For all subjects, published kinetic parameters were adjusted to reflect our current working-hypothesis compartmental model for whole-body vitamin A metabolism, which is shown in **Supplemental Figure 1**.

### Kinetic data and prediction of TBS by RID

Using the kinetic parameters and plasma retinol pool size for each subject [**Supplemental Tables 1** (adults) and **2** (children)], we created decks (i.e., modeling code) in WinSAAM, the Windows version of the Simulation, Analysis and Modeling software [www.winsaam.org (23–25)]. The decks were set up in light of an 8-component compartmental model (Supplemental Figure 1), adapted from reference 9, that describes whole-body vitamin A kinetics in humans following ingestion of stable isotope-labeled retinyl acetate on day 0 (see **Supplemental WinSAAM Deck**). Using WinSAAM, we simulated tracer responses (fraction of dose) in all compartments over 30 d for each subject, focusing on the terms in the RID prediction equation of Green et al. (16), which is shown here as Equation 1:

(1) $$\begin{eqnarray*}
{\rm{TBS}}\,\left( {{\rm{\mu mol}}} \right)\, = Fa \times S \times \,1/{\rm{S}}{{\rm{A}}_{\rm{p}}}\end{eqnarray*}$$

where, at any time *t*, *Fa*, the fraction of dose in stores, is calculated as the sum of the fraction of dose in the 2 storage compartments (Supplemental Figure 1); *S* is SA_p_/SA_s_, with SA_p_ equal to the fraction of dose in plasma (FD_p_) divided by the plasma retinol pool size (micromoles), and SA_s_ is calculated by dividing *Fa* by the amount of vitamin A (micromoles) in the 2 storage compartments (i.e., TBS). As noted earlier, SA_p_ is the measured variable for each individual in a RID study. After solving the differential equations derived for each deck in WinSAAM, values for *Fa*, *S*, and SA_p_, as well as the assigned values for TBS (i.e., sum of vitamin A in the 2 storage compartments determined using a steady-state solution in WinSAAM), were calculated separately for adults and children; values for *Fa* and *S* were tabulated as the composite coefficient *Fa* × *S* (i.e., *FaS*). We grouped data for adults and children separately in light of previous work showing that vitamin A kinetics are different in children and adults (9, 10, 26), as well as the assumption that, within a population, stores in children will likely be smaller than those in adults and the likelihood that field studies would focus on one group or the other.

Using the tabulated data, we calculated individual subject values for the composite RID coefficient *FaS* on days 1 through 30 as well as the geometric mean, SD, and CV% for each group. Then we used the geometric mean *FaS* and individual subject values for SA_p_ in Equation *1* to predict TBS in individuals over time; we also calculated group geometric mean values and ranges, as well as rank order (low to high), for RID-predicted TBS. Geometric mean and individual subject predictions for TBS at various times were compared with assigned values for TBS.

### Data management and statistics

Data are presented as geometric means, with ranges or SD. Microsoft Excel was used for data manipulation and calculations, and GraphPad Prism was used to develop figures and for regression analyses. *P* < 0.05 was considered significant.

## Results

Individual subjects’ assigned values for kinetic parameters and TBS are shown in Supplemental Tables 1 (adults) and 2 (children). As summarized in **Table 1**, assigned values for TBS ranged from 160 to 2734 μmol in the 20 theoretical adults (geometric mean = 641 μmol), and from 29.0 to 1107 (geometric mean = 284) μmol in the 20 children.

**FIGURE 1:**
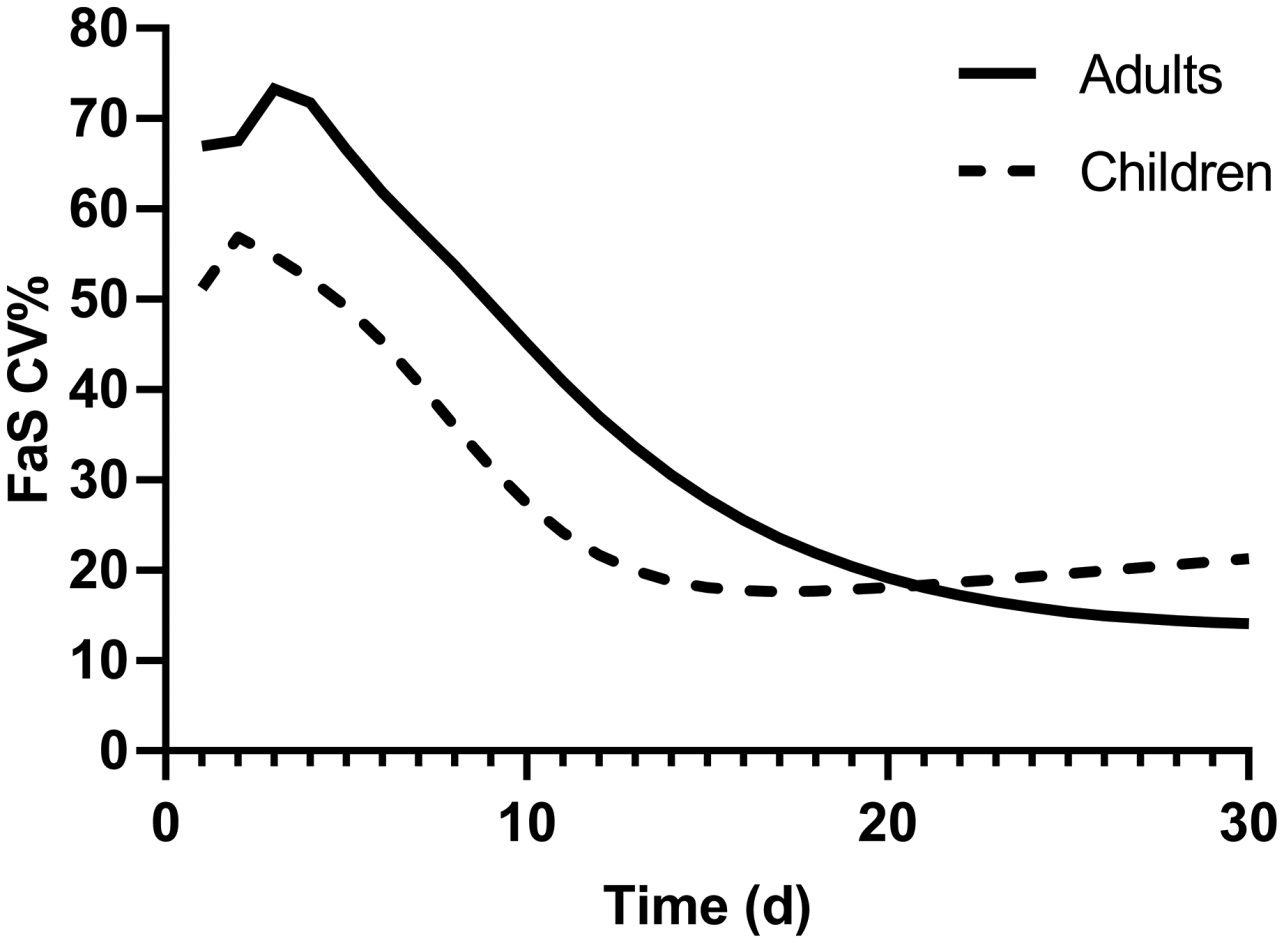
CV for the RID coefficient *FaS* over time in theoretical adults and children. Shown is CV% for 20 theoretical adults (Supplemental Table 1) and 20 theoretical children (Supplemental Table 2) for 30 d after ingestion of stable isotope-labeled retinyl acetate on day 0; the RID coefficient *FaS* was calculated as described in Methods, and geometric means were computed at each time. RID, retinol isotope dilution.

**TABLE 1:** Assigned and RID-predicted TBS in theoretical subjects^1^

		Predicted					
	Assigned	Day 4	Day 7	Day 10	Day 14	Day 21	Day 28
Adults, μmol							
Gmean	641	641	641	641	641	641	641
Min	160	235	171	157	160	173	186
Max	2734	3328	2318	1945	1767	1961	2268
Children, μmol							
GMean	284	284	284	284	284	284	284
Min	29.0	19.6	23.7	32.1	43.1	52.1	55.5
Max	1107	754	1017	1109	1038	1034	1049

1 Shown are GMean, Min, and Max assigned values for TBS and for RID predictions of TBS at various times after theoretical subjects [20 adults (Supplemental Table 1) and 20 children (Supplemental Table 2)] ingested a dose of stable isotope-labeled retinyl acetate. The RID prediction equation is presented in Methods as Equation *1*. GMean, geometric mean; Max, maximum; Min, minimum; RID, retinol isotope dilution; TBS, total body stores.

Using WinSAAM and assigned kinetic parameters and state variables, we simulated values for the RID equation coefficients *Fa* and *S* for each individual. Geometric mean values and SD for *FaS* in the 2 groups, as well as for all subjects combined, are listed in **Supplemental Table 3** for each day (1 to 30), and values for days 4, 7, 10, 14, 21, and 28 are summarized in **Table 2**; the first 5 of those 6 times have been used or considered for RID studies. Values for *FaS* decreased with time in both groups (**Supplemental Figure 2**) and were on average 12% lower in the children at all times. For both groups, the temporal decline in *FaS* followed a perfect biexponential function (*R*^2 ^= 1). In light of earlier findings (6, 16) that the most accurate RID predictions of TBS will be obtained when the CV for *FaS* is lowest, we plotted group mean values for CV% over time (**Figure 1**). The peak CV (71%) for *FaS* in this group of adults was observed on day 3; this was followed by a nearly perfect double-exponential decline to 29% on day 14, 17% on day 21, and 14% on day 30. For this group of children, CV was also high initially (60% at 2 d) but then it fell to 20% on day 14, with a nadir of 19% on day 18, and then a slow rise to 21% at day 30. Because we showed (16) that SA_p_ was the term in Equation *1* that had the highest predictive value for TBS, we regressed assigned TBS and 1/SA_p_. Results showed that, in the adults, the coefficient of determination (*R*^2^) significantly increased with time, from 0.37 at day 4 to 0.80 at day 14, 0.96 at day 21, and 0.99 at day 28; for the children, corresponding values were 0.77, 0.94, 0.97, and 0.98. When TBS was regressed with *FaS*, there were smaller but significant correlations, with *R*^2^ increasing for the adults from 0.28 at day 4 to 0.55 at day 28, and from 0.21 to 0.34, respectively, in the children.

**TABLE 2:** Values for the RID coefficient *FaS* over time in theoretical subjects^1^

	Adults		Children	
Time, d	GMean	SD	GMean	SD
4	1.83	1.28	1.51	0.798
7	0.987	0.564	0.914	0.372
10	0.809	0.352	0.731	0.205
14	0.727	0.209	0.642	0.130
21	0.658	0.110	0.588	0.113
28	0.621	0.0850	0.562	0.117

1 Shown are geometric means and SD for the composite coefficient *FaS* at 6 timepoints after theoretical subjects [20 adults (Supplemental Table 1) and 20 children (Supplemental Table 2)] ingested stable isotope-labeled retinyl acetate on day 0; data for each day from 1 to 30 are shown in Supplemental Table 3. For the RID equation used here (Equation *1*, see Methods), *Fa* is fraction of the dose in stores at time *t* and *S* is the ratio of specific activity of retinol in plasma to that in stores. GMean, geometric mean; RID, retinol isotope dilution.

In addition to assigned values, Table 2 also includes RID-predicted geometric means and ranges for TBS on days 4, 7, 10, 14, 21, and 28. Note that the predicted means are the same as the assigned values at all times for both groups, showing that when the appropriate value for the composite coefficient *FaS* was used in RID Equation *1*, the correct value for group mean TBS was obtained at any time from 1 to 30 d. For this group of theoretical adults, the range for predicted TBS (2429 μmol) was closest to that for assigned TBS (2574 μmol) at 6 d, and for children, corresponding ranges (1077 and 1078 μmol, respectively) were closest at 10 d, with variable but informative ranges obtained at all times for both groups (**Supplemental Figure 3**). Based on these results, we conclude that if the RID method is applied at 7 or 10 d, one not only obtains an accurate estimate of mean TBS for the group, but also reasonable estimates of the range.

**FIGURE 2:**
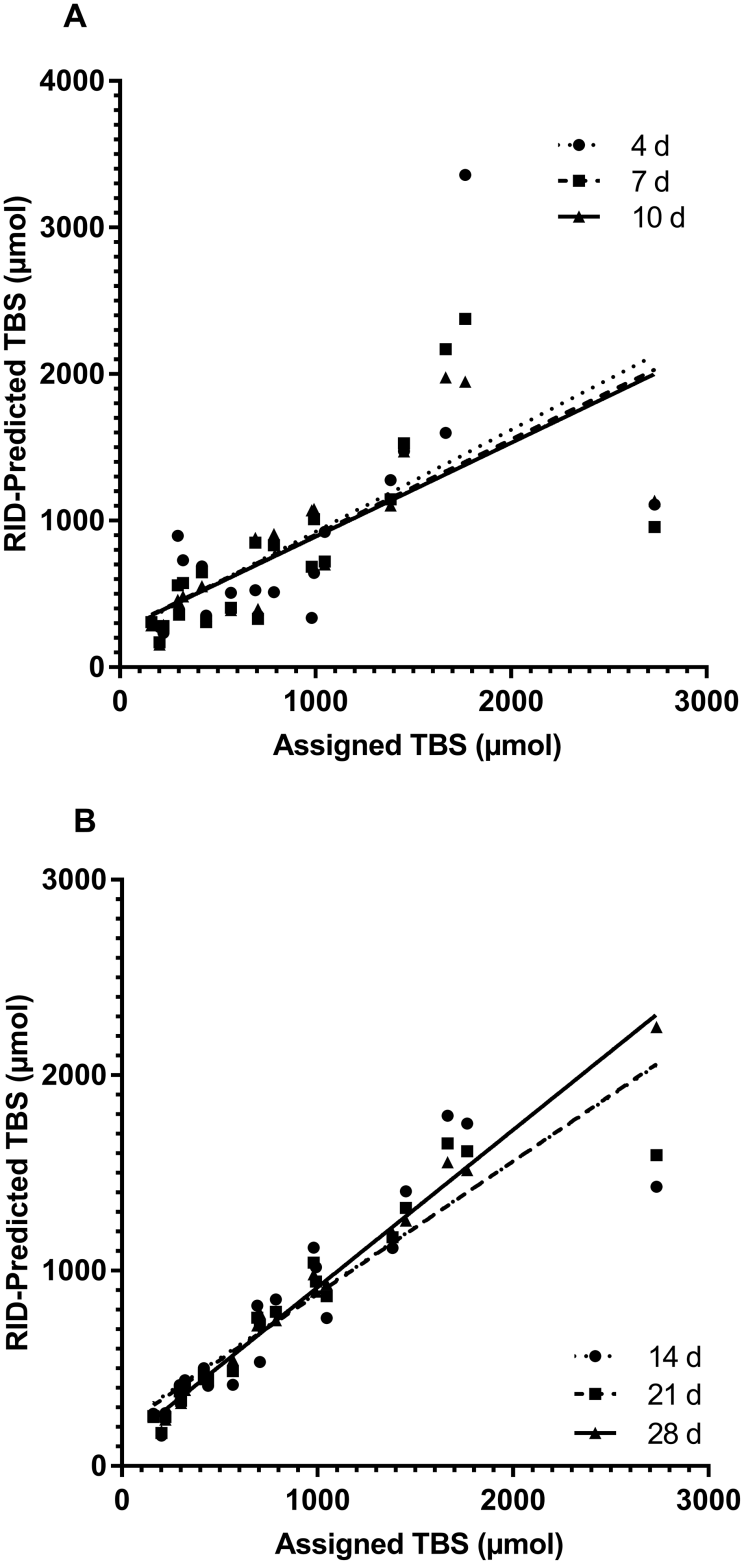
Assigned compared with RID-predicted TBS at 4, 7, and 10 d (A) and at 14, 21, and 28 d (B) in 20 theoretical adults. Shown are assigned (Supplemental Table 1) and predicted values for TBS after subjects ingested stable isotope-labeled retinyl acetate on day 0; RID Equation *1* (see Methods) was applied to predict TBS at the 6 times indicated. Regression equations are: *y* = 0.650*x* + 263 (*R*^2^ = 0.370, *P *= 0.0044) at day 4; *y* = 0.640*x* + 260 (*R*^2^ = 0.516, *P *= 0.0004) at day 7; *y* = 0.636*x* + 253 (*R*^2^ = 0.636, *P* < 0.0001) at day 10; *y* = 0.672*x* + 213 (*R*^2^ = 0.800, *P* < 0.0001) at day 14; *y* = 0.752*x* + 148 (*R*^2^ = 0.962, *P* < 0.0001) at day 21; and *y* = 0.805*x* + 109 (*R*^2^ = 0.992, *P* < 0.0001) at day 28. RID, retinol-isotope dilution; TBS, total body stores.

**FIGURE 3:**
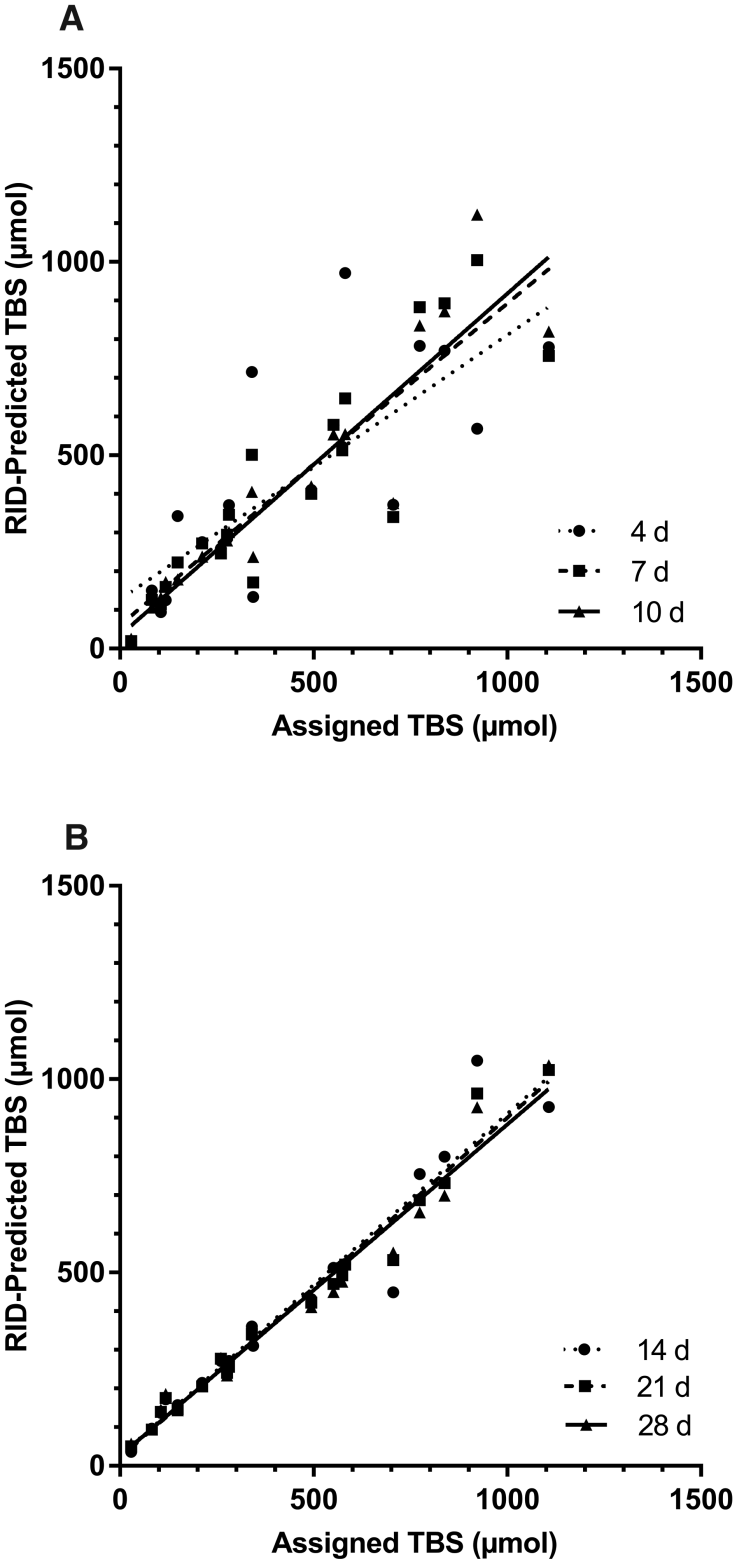
Assigned compared with RID-predicted TBS at 4, 7, and 10 d (A) and at 14, 21, and 28 d (B) in 20 theoretical children. Shown are assigned (Supplemental Table 2) and predicted values for TBS after subjects ingested stable isotope-labeled retinyl acetate on day 0; RID Equation *1* (see Methods) was applied to predict TBS at the 6 times indicated. Regression equations are: *y* = 0.588*x* + 123 (*R*^2^ = 0.768, *P* < 0.0001) at day 4; *y* = 0.784*x* + 58.5 (*R*^2^ = 0.825, *P* < 0.0001) at day 7; *y* = 0.851*x* + 34.1 (*R*^2^ = 0.870, *P* < 0.0001) at day 10; *y* = 0.868*x* + 24.9 (*R*^2^ = 0.940, *P* < 0.0001) at day 14; *y* = 0.876*x* + 19.5 (*R*^2^ = 0.968, *P *< 0.0001) at day 21; and *y* = 0.860*x* + 22.3 (*R*^2^ = 0.968, *P *< 0.0001) at day 28. RID, retinol-isotope dilution; TBS, total body stores.

Correlations between assigned values and RID-predicted TBS for individual adults and children are presented in **Figures 2** and **3**, respectively. Results for the 20 theoretical adults (Figure 2) show that there was a highly significant correlation between assigned and RID-predicted TBS, with *R*^2^ increasing from 0.370 at day 4 to 0.992 at day 28; the plot for this group indicates that day 21 or 28 would be an optimal time to do the RID test in adults like these. When values for TBS were ranked for adults from lowest to highest (**Supplemental Figure 4**), there was a highly significant correlation at all times, with *R*^2^ increasing from 0.534 on day 4 to 0.915 on day 14, 0.967 on day 21, and 0.970 on day 28. Qualitatively similar results for assigned compared with RID-predicted TBS were obtained for the 20 theoretical children (Figure 3). Specifically, assigned and predicted values were very close by day 14 (*R*^2 ^= 0.940), with just marginal improvement at days 21 and 28 (*R*^2 ^= 0.968), indicating that days 14 to 28 would be appropriate times to apply RID. As with the adults, when TBS values for the children were ranked (**Supplemental Figure 5**), there was a highly significant correlation at all times, with *R*^2^ increasing from 0.692 at day 4 to 0.97 at day 14, 0.98 at day 21, and 0.96 at day 28.

**FIGURE 4:**
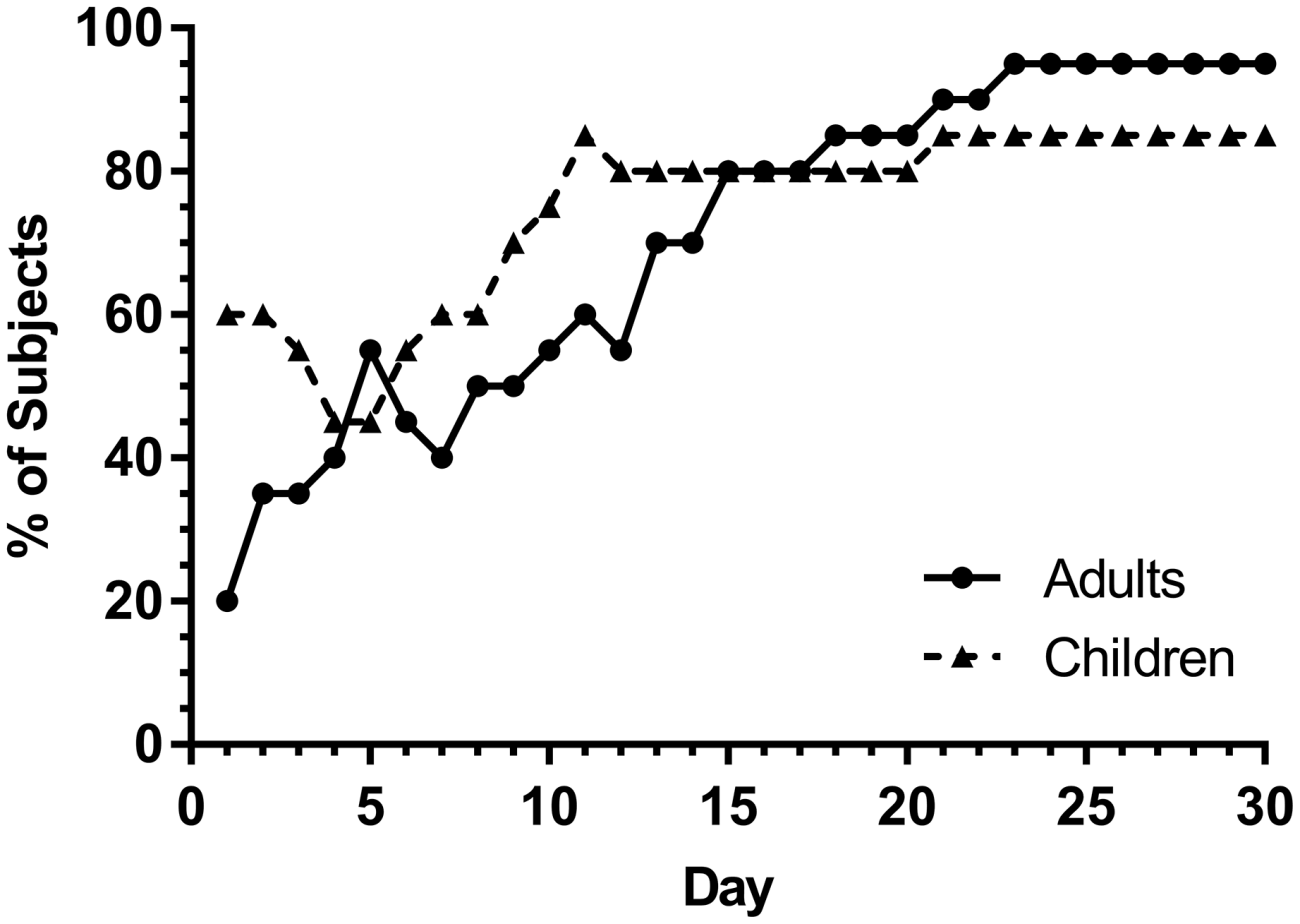
Percentage of theoretical subjects whose RID-predicted TBS was within 25% of assigned TBS versus time. Assigned values for TBS are shown for 20 theoretical adults and 20 theoretical children in Supplemental Tables 1 and 2, respectively. RID Equation *1* was used to predict TBS as described in Methods for 1–30 d after ingestion of stable isotope-labeled retinyl acetate on day 0. RID, retinol isotope dilution; TBS, total body stores.

**FIGURE 5:**
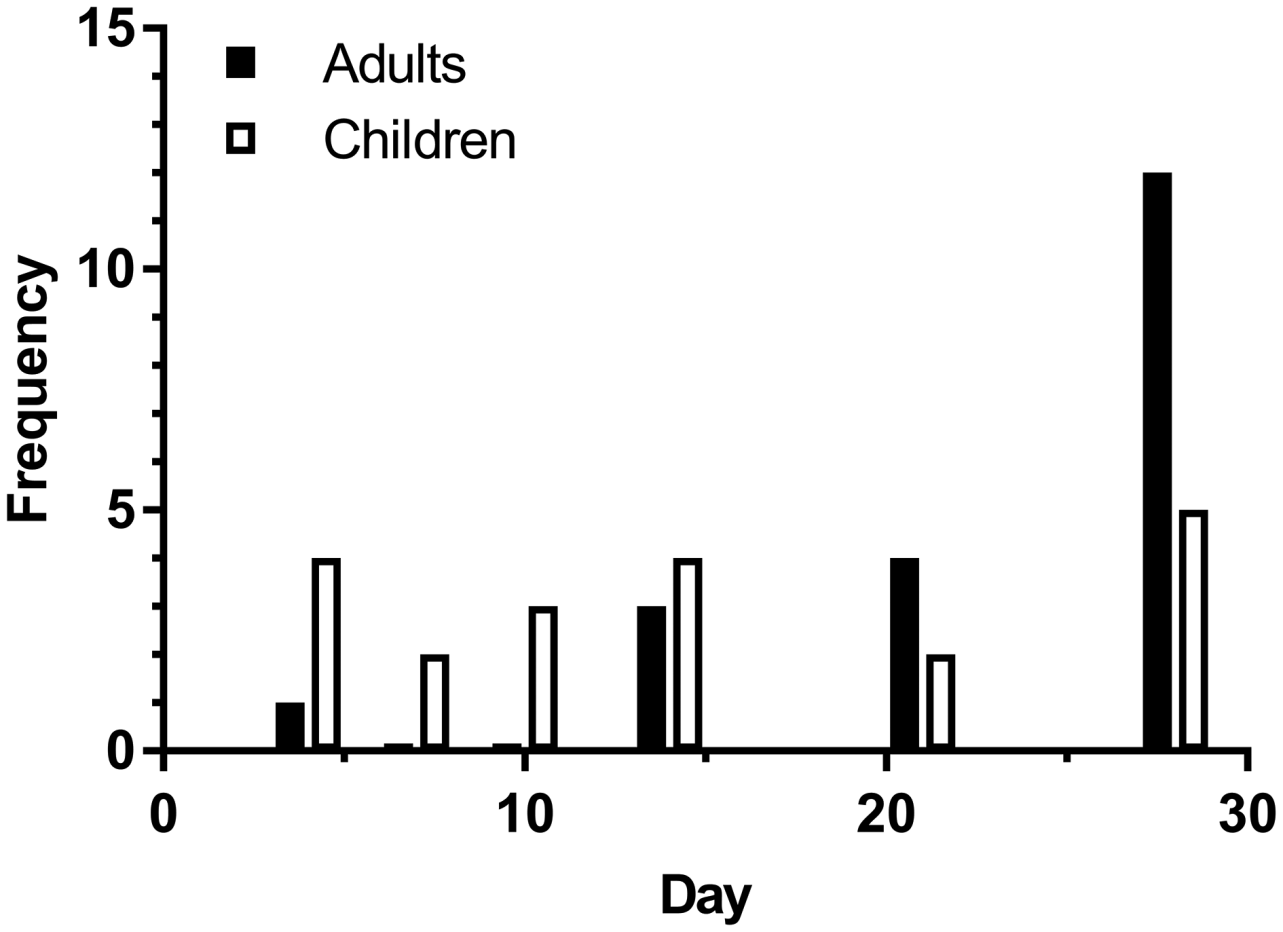
Frequency of most accurate prediction of TBS by RID at 6 selected times in theoretical subjects. RID Equation *1* was used to predict TBS as described in Methods at 4, 7, 10, 14, 21, and 28 d after ingestion of stable isotope-labeled retinyl acetate on day 0 by 20 theoretical adults and 20 theoretical children whose assigned values for TBS are listed Supplemental Tables 1 and 2, respectively; total frequency equals 20 for each group. RID, retinol isotope dilution; TBS, total body stores.

To further examine the accuracy of RID-predicted TBS at different times, we calculated the percentage of individuals within each group whose predicted values for TBS were within 10%, 25%, or 50% of assigned values at various times after dosing (**Supplemental Table 4**), with a summary in **Figure 4** of the results for 25%, the criterion we have used previously (5). For the 20 theoretical adults, there was a steady rise from 20% of individuals having a predicted TBS within 25% of their assigned value at day 1 to 80% at day 15 and 95% from days 23 to 30. For the children, predictions for 60% of subjects were within 25% of assigned TBS at day 1 and then, following a dip at days 4 and 5, the percentage rose to 85% on day 11, it was 80% from days 12 to 20, and it increased again to 85% for days 21 to 30. We also determined on which of 6 selected days RID most accurately predicted the assigned TBS in the most subjects (**Figure 5**). For the adults, the best time was day 28, followed by day 21 and then day 14; for the children, the best day was more evenly distributed, as shown, with day 28 best for the most children, followed by days 14, 4, and 10. In summary, assuming as we did here that individual subject values for the composite RID coefficient *FaS* are not available, our results indicate that no one time was best for predicting TBS in every subject within each group but that accurate predictions for a high proportion of subjects could be obtained at specific times (15 to 30 d for adults and 11 to 30 d for children).

## Discussion

Here, we applied compartmental modeling to simulated data for hypothetical adults and children to determine optimal blood sampling time(s) for estimating TBS by RID in both a group and its individuals. The use of theoretical subjects, as we have done previously (5, 19–22) enabled us to evaluate the accuracy of our findings by comparing RID predictions of TBS with assigned (known) values; inclusion of subjects with a wide range of values for TBS (<200 to >2500 μmol) reflects variations in vitamin A status that have been reported in field studies (e.g., 9, 27). Our results show that if we used model-simulated group mean values for the composite RID coefficient *FaS*, along with individual subject values for SA_p_, to predict TBS with RID Equation *1*, we obtained the correct group mean TBS at any time between 1 and 30 d after isotope dosing. Note that if one were calculating *FaS* based on modeling a composite dataset obtained using a super-subject approach, as was done by Ford et al. (9) in children in 3 lower-income countries, RID will predict a constant group mean TBS at all times if the model-generated SA_p_ (as opposed to the observed group mean SA_p_) is used in the RID equation. The values provided for *FaS* versus time (Table 2, Supplemental Table 3) could be useful in future RID studies.

Although our observations related to predicting group mean TBS are important, our main goal here was to determine the accuracy of results for individuals at various times. To do so, we evaluated correlations between assigned and predicted TBS (Figures 2 and 3), the rank order of predictions (Supplemental Figures 3 and 4), the percentage of individuals whose RID-predicted TBS was within 25% of the assigned value on a given day (Figure 4, with data for 10% and 50% also included in Supplemental Table 4), and the frequency at which specific days provided the most accurate prediction of TBS for individuals (Figure 5). Although very accurate predictions were obtained for many individuals in each group at numerous times, the highest percentage of adults predicted to be within 25% of their assigned TBS was at 20 to 28 d after dose administration, and for the children, it was at ≥10 d (Figure 4). Although it was disappointing to find both that early sampling times did not provide the best results and that there was no single time that was best for all individuals, it was nonetheless encouraging that several times were equally accurate for a large percentage of individuals in both groups.

Our results raise several interesting practical points. First, given the typical goals of RID studies done in community settings, it is important for researchers to specify the level of accuracy deemed acceptable for estimating TBS in individual subjects. In most instances, it is likely sufficient to estimate an individual's TBS within 25% of the actual value. If we extrapolate current results, this means that TBS for a vast majority of individuals would likely be accurately predicted at a number of different times. Secondly, our results (Figure 2) showing that the agreement between assigned and predicted TBS is better for lower values than higher ones are important because it is individuals in the former range that are of interest for vitamin A intervention. Finally, our current results suggest that if researchers are using a super-subject design as part of an RID study [as in Ford et al. (9)], accuracy for a given individual can be improved if reported TBS is based on analysis of 2 blood samples rather than 1. As currently used, and assuming a study length of 42 d and a sample size of 60, all subjects are sampled at a common time (4 d in reference 9) and each is also sampled at 1 additional time (5 subjects/time). However, our current data suggest that it might be better to sample 5 subjects at many of the selected times but 20 individuals at several later times (e.g., 14, 21, and 28 d) to improve identifiability for model parameters and thus *FaS*, providing better results for the majority of subjects.

Although a sampling time of ∼21 d after dosing was originally used for application of the RID method by Furr et al. (2), researchers have wondered if an earlier time might provide good predictions of TBS. In fact, studies in rats (15, 28, 29) indicated that 3 d was an optimal time to apply RID in this species, and Green (11) presented a parallel case in which it was recommended that the method be applied at 4 or 5 d in young adults (16). The advantages of using an earlier sampling time are that fraction of dose in plasma is still high (enhancing accuracy for quantification) and it is easier to retain subjects and maximize the chance that their health status will be the same as it was at the time of dosing. Our current results indicate that those potential advantages are outweighed by the finding that although the group mean value for stores can be accurately predicted by Equation *1* at any time from 1 to 30 d, the most accurate RID predictions of TBS for the most individuals were not obtained until ≥2 wk after dosing. Later times have the additional advantage that the CV for *FaS* is lower than at early times (Figure 1), leading to more accurate predictions of TBS in individual subjects (6).

For this analysis, we chose theoretical subjects who had a wide range of values for TBS (160–2734 μmol for the 20 adults and 29–1107 μmol for the children). One would expect that the range of values might be narrower in a typical field study if a relatively homogeneous population is intentionally selected. In that case, the CV for the RID coefficient *FaS* would likely be lower than what we found for these theoretical subjects, and consequently TBS predictions for individuals would likely be even more accurate than here; values might also be more accurate at earlier times, especially in children. Overall, predictions of TBS should be quite accurate at most times for subjects whose value for *FaS* is near the geometric mean.

The use of modeling to estimate *FaS* over time for a group, as we did here, represents an improvement over the alternative choice of using values obtained from the literature; in fact, if researchers planned an RID study but were unable to include the super-subject modeling aspect, they could use appropriate *FaS* values from those presented here rather than estimates proposed in 1989 (2). As shown in Table 1 and Supplemental Table 3, it is worth emphasizing that *FaS* varies with time and, in addition, patterns for individuals vary uniquely (data not shown). In fact, reviewing results from several studies in this laboratory, it was noted (JL Ford and MH Green, 2020, unpublished observations) that individual subject plots for *FaS* over time parallel response curves for FD_p_. In fact, if we expand and rearrange Equation *1*, we find that *FaS*/FD_p _= TBS/M(5), where M(5) is plasma retinol pool size (see Supplemental Figure 1), thus revealing why the curves are parallel. The fact that, at any given time, individuals have unique values for both FD_p_ and *FaS* explains why we could not identify a single time that provided the most accurate prediction of TBS for all individuals in each group. However, as we have previously reported (6), the closer the group geometric mean *FaS* is to an individual's value for the composite coefficient, the more accurate will be the prediction of that subject's TBS, emphasizing the importance of applying the method at times when the CV% for *FaS* is lowest. In addition, it is important to point out that the variation in *FaS* is not primarily related to variation in *Fa*, because the range of values for tracer absorption in healthy individuals is presumably relatively narrow (e.g., 65–85%) and because, within several days after dosing, essentially all the absorbed tracer will be found in stores. In contrast, because the variability in *S* is due to the variability in both SA_p_ and SA_s_, and both are related to the variability in TBS, it is this factor that is the primary contributor to variation in the composite coefficient. Importantly, in the context of the RID prediction equation and as shown by Green et al. (16) and confirmed with the current data, it is SA_p_, the measured variable, that is the strongest predictor of TBS in individual subjects. Thus, by simply evaluating the measured variable for a given individual within a group, one has a good of idea of that subject's vitamin A status relative to others in the group.

In conclusion, the current work further demonstrates the usefulness of applying compartmental modeling to data for theoretical subjects as a means to evaluate and improve the RID technique for estimating vitamin A TBS. In conjunction with a super-subject study or by using results from an appropriate theoretical or prior field study, RID can provide accurate estimates of group mean TBS and reasonably accurate predictions for individual subjects at various times.

## Supplementary Material

nxab061_Supplemental_File

## Notes

Support for this work was provided by the Bill & Melinda Gates Foundation (Project Number OPP1115464).

Author disclosures: The authors report no conflicts of interest.

Supplemental Tables 1–4, Supplemental Figures 1–5, and Supplemental WinSAAM Deck are available from the “Supplementary data” link in the online posting of the article and from the same link in the online table of contents at http://academic.oup.com/jn.

Abbreviations used: FD_p_, fraction of dose in plasma; RID, retinol isotope dilution; SA_p_, retinol specific activity in plasma; SA_s_, retinol specific activity in stores; TBS, vitamin A total body stores; WinSAAM, Windows version of Simulation, Analysis and Modeling software.

## References

[1] Bausch J , Rietz P. Method for the assessment of vitamin A liver stores. Acta Vitaminol Enzym. 1977;31:99–112. 580694

[2] Furr HC , Amedee-Manesme O, Clifford AJ, Bergen HR 3rd, Jones AD, Anderson DP, Olson JA. Vitamin A concentrations in liver determined by isotope dilution assay with tetradeuterated vitamin A and by biopsy in generally healthy adult humans. Am J Clin Nutr. 1989;49:713–16. 2648799 10.1093/ajcn/49.4.713

[3] Lietz G , Furr HC, Gannon BM, Green MH, Haskell M, Lopez-Teros V, Novotny JA, Palmer AC, Russell RM, Tanumihardjo SA et al. Current capabilities and limitations of stable isotope techniques and applied mathematical equations in determining whole-body vitamin A status. Food Nutr Bull. 2016;37:S87–103. 27053491 10.1177/0379572116630642

[4] Hunt JR , Kurpad AV. Introduction to symposium proceedings “Applying Vitamin A Isotope Dilution Techniques to Benefit Human Nutrition.” Int J Vitam Nutr Res. 2014;84(Suppl 1):7–8. 25537100 10.1024/0300-9831/a000180

[5] Ford JL , Green JB, Green MH. A population-based (super-child) approach for predicting vitamin A total body stores and retinol kinetics in children is validated by the application of model-based compartmental analysis to theoretical data. Curr Dev Nutr. 2018;2:nzy071. 30488046 10.1093/cdn/nzy071 PMC6252344

[6] Green MH , Green JB, Ford JL. Better predictions of vitamin A total body stores by the retinol isotope dilution method are possible with deeper understanding of the mathematics and by applying compartmental modeling. J Nutr. 2020;150:989–93. 31851323 10.1093/jn/nxz321 PMC7198291

[7] Lopez-Teros V , Ford JL, Green MH, Tang G, Grusak MA, Quihui-Cota L, Muzhingi T, Paz-Cassini M, Astiazaran-Garcia H. Use of a “‘super-child’” approach to assess the vitamin A equivalence of *Moringa oleifera* leaves, develop a compartmental model for vitamin A kinetics, and estimate vitamin A total body stores in young Mexican children. J Nutr. 2017;147:2356–63. 28931584 10.3945/jn.117.256974

[8] Van Stuijvenberg ME , Dhansay MA, Nel J, Suri D, Grahn M, Davis CR, Tanumihardjo SA. South African preschool children habitually consuming sheep liver and exposed to vitamin A supplementation and fortification have hypervitaminotic A liver stores: a cohort study. Am J Clin Nutr. 2019;110:91–101. 31089689 10.1093/ajcn/nqy382

[9] Ford JL , Green JB, Haskell MJ, Ahmad SM, Mazariegos Cordero DI, Oxley A, Engle-Stone R, Lietz G, Green MH. Use of model-based compartmental analysis and a super-child design to study whole-body retinol kinetics and vitamin A total body stores in children from 3 lower-income countries. J Nutr. 2020;150:411–18. 31535129 10.1093/jn/nxz225 PMC7004890

[10] Lopez-Teros V , Ford JL, Green MH, Monreal-Barraza B, García-Miranda L, Tanumihardjo SA, Valencia ME, Astiazaran-Garcia H. The “super-child” approach is applied to estimate retinol kinetics and vitamin A total body stores in Mexican preschoolers. J Nutr. 2020;150:1644–51. 32135013 10.1093/jn/nxaa048

[11] Green MH . Evaluation of the “Olson equation”, an isotope dilution method for estimating vitamin A stores. Int J Vitam Nutr Res. 2014;84:9–15. 25537101 10.1024/0300-9831/a000181

[12] Haskell MJ , Lembcke JL, Salazar M, Green MH, Peerson JM, Brown KH. Population-based plasma kinetics of an oral dose of [^2^H_4_]retinyl acetate among preschool-aged Peruvian children. Am J Clin Nutr. 2003;77:681–6. 12600861 10.1093/ajcn/77.3.681

[13] Ribaya-Mercado JD , Mazariegos M, Tang GW, Romero-Abal ME, Mena I, Solomons NW, Russell RM. Assessment of total body stores of vitamin A in Guatemalan elderly by the deuterated-retinol-dilution method. Am J Clin Nutr. 1999;69:278–84. 9989693 10.1093/ajcn/69.2.278

[14] Tang GW , Qin J, Hao LY, Yin SA, Russell RM. Use of a short-term isotope-dilution method for determining the vitamin A status of children. Am J Clin Nutr. 2002;76:413–18. 12145015 10.1093/ajcn/76.2.413

[15] Green MH , Green JB, Lewis KC. Variation in retinol utilization rate with vitamin A status in the rat. J Nutr. 1987;117:694–703. 3585518 10.1093/jn/117.4.694

[16] Green MH , Ford JL, Green JB, Berry P, Boddy AV, Oxley A, Lietz G. A retinol isotope dilution equation predicts both group and individual total body vitamin A stores in adults based on data from an early postdosing blood sample. J Nutr. 2016;146:2137–42. 27511937 10.3945/jn.116.233676 PMC5037874

[17] Gannon BM , Tanumihardjo SA. Comparisons among equations used for retinol isotope dilution in the assessment of total body stores and total liver reserves. J Nutr. 2015;145:847–54. 25809683 10.3945/jn.114.208132 PMC6619684

[18] Mondloch S , Gannon BM, Davis CR, Chileshe J, Kaliwile C, Masi C, Rios-Avila L, Gregory JF, Tanumihardjo SA. High provitamin A carotenoid serum concentrations, elevated retinyl esters, and saturated retinol-binding protein in Zambian preschool children are consistent with the presence of high liver vitamin A stores. Am J Clin Nutr. 2015;102:497–504. 26178727 10.3945/ajcn.115.112383 PMC6546228

[19] Green MH , Ford JL, Green JB. Retinol isotope dilution is applied during restriction of vitamin A intake to predict individual subject total body vitamin A stores at isotopic equilibrium. J Nutr. 2016;146:2407–11. 27683870 10.3945/jn.116.238899

[20] Ford JL , Green JB, Green MH. Should we restrict vitamin A intake, a minor contributor to plasma retinol turnover, when using retinol isotope dilution equations to estimate an individual's vitamin A status, or should vitamin A balance be maintained?. J Nutr. 2017;147:1483–6. 28701388 10.3945/jn.117.254441

[21] Ford JL , Green JB, Green MH. Addition of vitamin A intake data during compartmental modeling of retinol kinetics in theoretical humans leads to accurate prediction of vitamin A total body stores and kinetic parameters in studies of reasonable duration. J Nutr. 2019;149:2065–72. 31187866 10.1093/jn/nxz112 PMC6825818

[22] Green MH , Green JB, Ford JL. Vitamin A absorption efficiency determined by compartmental analysis of postprandial plasma retinyl ester kinetics in theoretical humans. J Nutr. 2020;150:2223–9. 32614427 10.1093/jn/nxaa176 PMC7398788

[23] Berman M , Weiss MF. The SAAM manual. Washington (DC): US Government Printing Office, US DHEW (NIH); 1978.

[24] Wastney ME , Patterson BH, Linares OA, Greif PC, Boston RC. Investigating biological systems using modeling: strategies and software. San Diego (CA): Academic Press; 1999. p. 95–138.

[25] Stefanovski D , Moate PJ, Boston RC. WinSAAM: a Windows-based compartmental modeling system. Metabolism. 2003;52:1153–66. 14506622 10.1016/s0026-0495(03)00144-6

[26] Cifelli CJ , Green JB, Wang Z, Yin S, Russell RM, Tang G, Green MH. Kinetic analysis shows that vitamin A disposal rate in humans is positively correlated with vitamin A stores. J Nutr. 2008;138:971–7. 18424609 10.1093/jn/138.5.971

[27] Green MH , Ford JL, Green JB. Inclusion of vitamin A intake data provides improved compartmental model-derived estimates of vitamin A total body stores and disposal rate in older adults. J Nutr. 2019;149:1282–7. 31095324 10.1093/jn/nxz056 PMC6602889

[28] Adams WR , Green MH. Prediction of liver vitamin A in rats by an oral isotope dilution technique. J Nutr. 1994;124:1265–70. 8064375 10.1093/jn/124.8.1265

[29] Duncan TE , Green JB, Green MH. Liver vitamin A levels in rats are predicted by a modified isotope dilution technique. J Nutr. 1993;123:933–9. 8487104 10.1093/jn/123.5.933

