# Supplementary material for: Use of Model-Based Compartmental Analysis and Theoretical Data to Further Explore Choice of Sampling Time for Assessing Vitamin A Status in Groups and Individual Human Subjects by the Retinol Isotope Dilution Method
Source: J Nutr. 2021 Apr 8;151(7):2068–74. doi: 10.1093/jn/nxab061 (PMC8245873; doi:10.1093/jn/nxab061)
Supplement: nxab061_Supplemental_File [file nxab061_supplemental_file.pdf]

Use of Model-Based Compartmental Analysis and Theoretical Data to Further Explore Choice of Sampling Time for Assessing Vitamin A Status in Groups and Individual Human Subjects by the Retinol Isotope Dilution Method; Green MH; Online Supplementary Material

SUPPLEMENTAL TABLE 1 Assigned values for retinol kinetic parameters and state variables for 20 theoretical adults[1]

Subject #	1	2	3	4	5	6	7	8	9	10	11
Parameters											
L(2,1), d^{-1}	30	30	30	30	30	30	30	30	30	30	30
L(0,1), d^{-1}	24.2	22.5	19.5	15.1	12.3	11.7	10.5	9.99	9.00	7.55	4.95
L(3,2), d^{-1}	30	30	30	30	30	30	30	30	30	30	30
L(4,3), d^{-1}	1	1	1	1	1	1	1	1	1	1	1
L(5,4), d^{-1}	2.07	1.90	1.09	1.20	2.00	1.41	0.792	0.945	0.756	1.57	2.00
L(7,5), d^{-1}	9.92	4.11	5.02	7.00	9.67	4.55	5.21	3.91	8.21	6.20	3.00
L(5,7), d^{-1}	4.89	2.06	2.45	3.51	4.68	2.27	2.64	2.00	4.08	3.01	1.50
L(6,5), d^{-1}	2.01	5.80	5.34	1.98	3.52	3.00	2.81	8.00	4.19	11.0	3.21
L(5,6), d^{-1}	0.0635	0.101	0.0838	0.0457	0.0417	0.0210	0.0137	0.0236	0.0260	0.0305	0.0153
L(9,6), d^{-1}	0.00354	0.000971	0.00149	0.000951	0.00143	0.000998	0.000732	0.000597	0.000928	0.000510	0.000989
L(8,5), d^{-1}	0.106	0.0554	0.0931	0.0404	0.116	0.136	0.142	0.198	0.144	0.180	0.194
L(11,8), d^{-1}	1	1	1	1	1	1	1	1	1	1	1
DT(3), d	0.0211	0.0211	0.0246	0.0280	0.0263	0.0279	0.0346	0.0235	0.0226	0.0277	0.0246
DT(8), d	0.052	0.052	0.052	0.052	0.052	0.052	0.052	0.052	0.052	0.052	0.052
State variables											
M(5), µmol	5	5	5	5	5	5	5	5	5	5	5
M(6), µmol	150	285	313	212	409	682	970	1655	778	1765	983
M(7), µmol	10.1	9.98	10.2	9.97	10.3	10.0	9.88	9.79	10.1	10.3	10.0
TBS, µmol	160	295	323	222	419	693	980	1665	788	1775	993
U(1), µmol/d	1.92	0.970	1.54	0.607	1.65	1.89	1.92	2.64	1.88	2.25	2.26
% Abs	55.3	57.0	60.6	66.5	70.9	71.9	74.0	75.0	76.9	79.9	85.8

SUPPLEMENTAL TABLE 1, continued

Subject #	12	13	14	15	16	17	18	19	20	GMean
Parameters										
L(2,1), d^{-1}	30	25	40	50	30	45	30	25	50	32.1
L(0,1, d^{-1})	3.29	8.32	13.3	16.6	9.99	15.0	9.99	8.32	16.6	11.2
L(3,2), d^{-1}	30	25	40	50	30	45	30	25	50	32.1
L(4,3), d^{-1}	1	1	1	1	1	1	1	1	1	1
L(5,4), d^{-1}	1.20	1.60	1.40	0.900	1.10	1.70	0.700	1.20	0.600	1.22
L(7,5, d^{-1})	0.984	9.00	5.00	4.00	3.50	14.0	5.50	3.00	4.50	5.07
L(5,7), d^{-1}	0.491	0.300	1.00	0.180	0.200	0.900	0.150	0.400	0.240	1.09
L(6,5), d^{-1}	6.50	2.00	4.00	2.80	3.50	5.00	6.00	2.75	5.60	3.98
L(5,6), d^{-1}	0.0212	0.0282	0.0578	0.0180	0.0192	0.0315	0.0261	0.0132	0.0105	0.0284
L(9,6), d^{-1}	0.00129	0.00325	0.00275	0.00300	0.00125	0.00150	0.00110	0.00165	0.000500	0.00125
L(8,5), d^{-1}	0.371	0.206	0.182	0.400	0.213	0.227	0.243	0.306	0.254	0.166
L(11,8), d^{-1}	1	1	1	1	1	1	1	1	1	1
DT(3), d	0.0208	0.220	0.240	0.110	0.160	0.230	0.170	0.190	0.210	0.0558
DT(8), d	0.052	0.052	0.052	0.052	0.052	0.052	0.052	0.052	0.052	0.052
State variables										
M(5), µmol	5.00	2.18	4.24	3.65	5.57	2.64	5.38	3.65	5.18	4.51
M(6), µmol	1442	138	280	487	951	400	1187	678	2637	601
M(7), µmol	10.0	65.2	21.2	81.1	97.5	41.1	197	27.4	97.1	20.9
TBS, µmol	1452	203	302	568	1048	441	1384	706	2734	642
U(1), µmol/d	4.12	1.20	2.06	3.89	3.17	1.60	3.48	2.98	3.52	2.06
% Abs	90.1	75.0	75.0	75.0	75.0	75.0	75.0	75.0	75.0	72.7

[1] Shown are assigned values (and geometric means) for vitamin kinetic parameters and state variables for 20 theoretical adults. Kinetic parameters listed are fractional transfer coefficients [L(I,J)s, or the fraction of retinol in compartment J transferred to compartment I each day] and delay times [DT(I)s, or the time (d) spent in delay component I]; state variables are masses [M(I)] of vitamin A in compartments 5, 6, 7, and in TBS (compartments 6 + 7), dietary vitamin A intake [U(1)], and vitamin A absorption efficiency (% Abs). The model is shown in Supplemental Figure 1. Subjects 1 – 12 are from (22) and subjects 12 – 20 are Y5 – Y8 and O9 – O12 from (21). GMean, geometric mean; TBS, total body stores.

SUPPLEMENTAL TABLE 2 Assigned values for retinol kinetic parameters and state variables for 20 theoretical children[1]

Subject #	1	2	3	4	5	6	7	8	9	10	11
Parameters											
L(2,1), d^{-1}	25.0	35.0	50.0	40.0	11.0	12.2	8.67	14.5	14.8	11.5	16.5
L(0,1), d^{-1}	8.32	11.6	16.6	13.3	3.46	4.66	2.40	2.19	4.15	1.46	2.79
L(3,2), d^{-1}	25.0	35.0	50.0	40.0	11.0	12.2	8.67	14.5	14.8	11.5	16.5
L(4,3), d^{-1}	1	1	1	1	1	1	1	1	1	1	1
L(5,4), d^{-1}	2.00	2.50	3.00	1.30	6.44	7.67	8.65	6.25	3.20	4.56	3.06
L(7,5), d^{-1}	12.0	10.0	40.0	20.0	30.0	25.7	23.4	20.0	18.7	15.6	26.5
L(5,7), d^{-1}	1.50	0.800	1.20	0.400	2.18	4.32	2.18	1.26	0.676	1.22	1.98
L(6,5), d^{-1}	2.50	6.80	30.0	22.0	15.8	12.5	25.7	13.0	12.8	17.2	14.1
L(5,6), d^{-1}	0.0225	0.0122	0.0306	0.0215	0.0846	0.104	0.0691	0.0351	0.0386	0.0524	0.0614
L(9,6), d^{-1}	0.00750	0.00118	0.000650	0.00150	0.00606	0.00258	0.00371	0.00822	0.00401	0.00380	0.00453
L(8,5), d^{-1}	0.625	0.598	0.623	1.43	1.05	0.303	1.31	2.46	1.21	1.16	0.967
L(11,8), d^{-1}	1	1	1	1	1	1	1	1	1	1	1
DT(3), d	0.250	0.180	0.150	0.200	0.216	0.154	0.154	0.214	0.214	0.221	0.228
DT(8), d	0.052	0.052	0.052	0.052	0.052	0.052	0.052	0.052	0.052	0.052	0.052
State variables											
M(5), µmol	0.320	0.660	0.845	1.10	0.437	1.21	0.374	0.374	0.793	0.884	0.936
M(6), µmol	26.7	336	810	1052	76.0	142	132	112	239	270	200
M(7), µmol	2.56	8.25	28.2	55.0	6.00	7.19	4.01	5.92	21.9	11.3	12.5
TBS, µmol	29.2	344	838	1107	82.0	149	136	118	260	281	212
U(1), µmol/d	0.533	1.05	1.40	4.21	1.21	1.01	1.25	2.12	2.45	2.31	2.11
% Abs	75.0	75.0	75.0	75.0	76.0	72.4	78.3	86.9	78.1	88.7	85.5

SUPPLEMENTAL TABLE 2, continued

Subject #	12	13	14	15	16	17	18	19	20	GMean
Parameters										
L(2,1)	12.2	11.1	8.88	15.8	8.36	12.0	12.4	10.5	13.6	14.9
L(0,1)	2.13	4.75	3.29	3.88	2.29	3.12	3.92	4.22	2.95	4.05
L(3,2)	12.2	11.1	8.88	15.8	8.36	12.0	12.4	10.5	13.6	14.9
L(4,3)	1	1	1	1	1	1	1	1	1	1
L(5,4)	5.87	4.74	11.9	4.45	4.36	3.00	4.00	2.80	4.60	4.15
L(7,5)	18.5	27.8	23.9	23.0	19.2	18.4	11.6	10.9	22.1	19.7
L(5,7)	1.17	2.17	1.22	0.682	0.696	0.571	0.800	0.148	1.20	1.06
L(6,5)	10.1	11.7	19.3	17.3	17.4	8.92	14.0	18.0	14.6	13.7
L(5,6)	0.0334	0.0164	0.0313	0.0324	0.0344	0.0197	0.0356	0.150	0.0452	0.0382
L(9,6)	0.00675	0.00115	0.000508	0.00135	0.00110	0.000721	0.000600	0.00164	0.00202	0.00208
L(8,5)	1.70	0.769	0.309	0.691	0.539	0.315	0.232	0.889	0.624	0.746
L(11,8)	1	1	1	1	1	1	1	1	1	1
DT(3)	0.195	0.224	0.199	0.197	0.170	0.227	0.216	0.178	0.217	0.198
DT(8)	0.052	0.052	0.052	0.052	0.052	0.052	0.052	0.052	0.052	0.052
State variables										
M(5)	0.391	1.35	1.23	0.905	1.11	1.50	1.37	0.945	0.844	0.795
M(6)	98.7	905	749	463	544	656	531	112	260	264
M(7)	6.21	17.4	24.1	30.5	30.6	48.5	19.9	69.5	15.5	14.8
TBS	105	922	774	494	574	705	551	182	276	284
U(1)	1.56	2.98	1.04	1.56	1.52	1.19	0.838	1.44	1.28	1.48
% Abs	85.2	70.1	73.0	80.3	78.5	79.4	76.0	71.4	82.2	77.9

[1] Shown are assigned values (and geometric means) for vitamin kinetic parameters and state variables for 20 theoretical children. Kinetic parameters listed are fractional transfer coefficients [L(I,J)s, or the fraction of retinol in compartment J transferred to compartment I each day] and delay times [DT(I)s, or the time (d) spent in delay component I]; state variables are masses [M(I)] of vitamin A in compartments 5, 6, 7, and in TBS (compartments 6 + 7), dietary vitamin A intake [U(1)], and vitamin A absorption efficiency (% Abs). The model is shown in Supplemental Figure 1. Subjects 1 – 4 are from (21) and 5 – 20 are adapted from (5). GMean, geometric mean; TBS, total body stores.

SUPPLEMENTAL TABLE 3 Values for the RID coefficient *FaS* over time in theoretical subjects[1]

Time (d)	Adults		Children		All	
	GMean	SD	GMean	SD	GMean	SD
1	8.42	5.51	6.66	3.78	7.49	4.84
2	4.61	3.09	2.93	1.75	3.68	2.70
3	2.73	1.99	1.99	1.12	2.33	1.67
4	1.83	1.32	1.51	0.798	1.66	1.10
5	1.37	0.925	1.22	0.604	1.29	0.781
6	1.13	0.708	1.04	0.470	1.08	0.599
7	0.987	0.578	0.914	0.372	0.950	0.484
8	0.902	0.487	0.831	0.300	0.866	0.404
9	0.847	0.418	0.773	0.246	0.809	0.343
10	0.809	0.361	0.731	0.205	0.769	0.296
11	0.781	0.314	0.699	0.176	0.739	0.258
12	0.760	0.275	0.676	0.155	0.716	0.227
13	0.742	0.242	0.657	0.141	0.698	0.202
14	0.727	0.214	0.642	0.131	0.683	0.182
15	0.713	0.191	0.630	0.124	0.670	0.166
16	0.702	0.171	0.620	0.119	0.660	0.152
17	0.691	0.155	0.612	0.116	0.650	0.142
18	0.681	0.141	0.605	0.114	0.642	0.133
19	0.673	0.130	0.598	0.114	0.635	0.126
20	0.665	0.121	0.593	0.113	0.628	0.121
21	0.658	0.113	0.588	0.113	0.622	0.116
22	0.651	0.106	0.584	0.113	0.616	0.113
23	0.645	0.101	0.580	0.114	0.611	0.110
24	0.640	0.0970	0.576	0.114	0.607	0.109
25	0.634	0.0936	0.572	0.115	0.602	0.107
26	0.630	0.0909	0.568	0.115	0.598	0.106
27	0.625	0.0888	0.565	0.116	0.594	0.106
28	0.621	0.0872	0.562	0.117	0.591	0.105
29	0.617	0.0860	0.558	0.118	0.587	0.105
30	0.614	0.0850	0.555	0.119	0.584	0.105

[1] Shown are GMean values, with SD, for the composite coefficient *FaS* versus time after ingestion of stable isotope-labeled retinyl acetate in 20 theoretical adults and 20 theoretical children and in all 40 subjects. *Fa* is fraction of dose in stores at time *t* and *S* is the ratio of specific activity of retinol in plasma to that in stores; see Equation *1* (Methods) for more details. Subjects were previously described in (2,24) (adults; Supplemental Table 1) and in (5,22) (children; Supplemental Table 2). *FaS* was calculated for the groups combined since values were less different than expected between the groups. GMean, geometric mean; RID, retinol isotope dilution.

SUPPLEMENTAL TABLE 4 Relative accuracy of RID-predicted TBS in theoretical subjects[1]

Time (d)	4	7	10	14	21	28
Adults						
10%	20	15	10	30	65	65
25%	40	40	55	70	90	95
50%	60	75	90	95	95	95
Children						
10%	25	30	40	50	45	45
25%	45	60	75	80	85	85
50%	80	95	100	100	95	90

[1] Data are % of subjects within each group of 20 whose RID-predicted TBS was within 10, 25, or 50% of the assigned value for TBS. Assigned values for TBS are shown in Supplemental Tables 1 (adults) and 2 (children). RID, retinol isotope dilution; TBS, total body stores.

SUPPLEMENTAL FIGURE 1

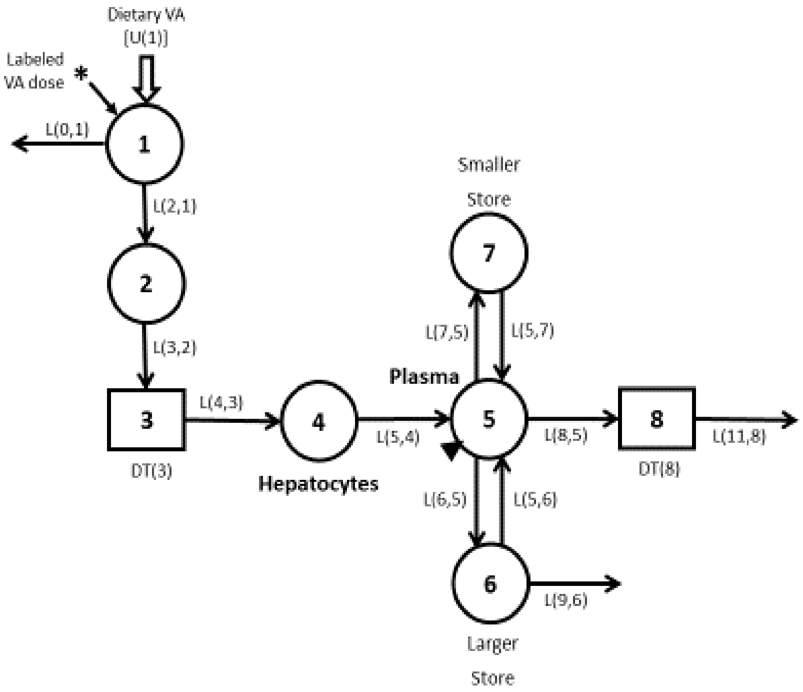

SUPPLEMENTAL FIGURE 1 Compartmental model for whole-body vitamin A metabolism in humans [adapted from (9)]. Circles represent compartments; the rectangles are delay elements; interconnectivities between components (arrows) are fractional transfer coefficients [L(I,J)s, or the fraction of retinol in compartment J transferred to compartment I each day] and delay times [DT(I)s, or days spent in delay element I]. Compartment 1 is the site of introduction of ingested tracer (*) and dietary vitamin A [U(1)]. Components 1 – 4 represent digestion, absorption, and chylomicron processing until uptake by hepatocytes (compartment 4), with subsequent secretion of retinol bound to retinol-binding protein into plasma compartment 5; compartment 5 is the site of sampling (triangle). Component 8 allows for irreversible uptake of plasma retinol by tissues from which retinol does not recycle. Retinol in plasma can also exchange with vitamin A in 2 extravascular pools (a larger compartment 6 and a smaller compartment 7), with compartments 6 and 8 the sites of irreversible loss from the system.

SUPPLEMENTAL FIGURE 2

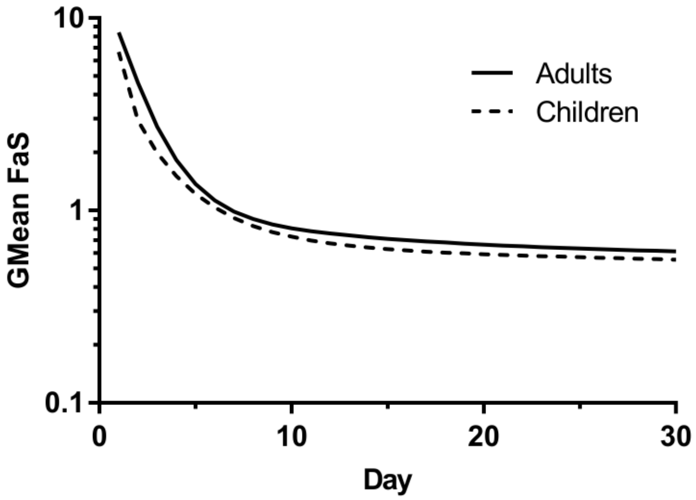

SUPPLEMENTAL FIGURE 2 The composite RID coefficient *FaS* versus time in theoretical subjects. Shown are GMean values for *FaS* over 30 d after ingestion of stable isotope-labeled retinyl acetate on d 0 by 20 theoretical adults (Supplemental Table 1) and 20 children (Supplemental Table 2). Values for *FaS* (Supplemental Table 3) were calculated as described in Methods; in brief, *Fa* is fraction of dose in stores at time *t* and *S* is the ratio of specific activity of retinol in plasma to that in stores. GMean, geometric mean; RID, retinol isotope dilution.

SUPPLEMENTAL FIGURE 3

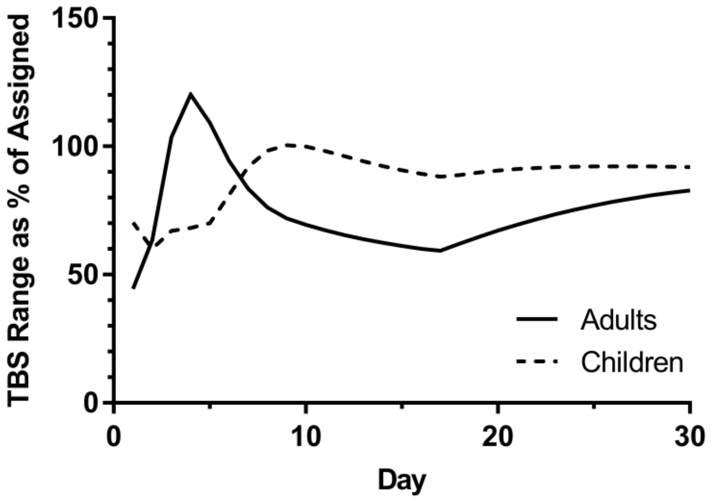

SUPPLEMENTAL FIGURE 3 Range for RID-predicted TBS as percent of assigned TBS range over time in 20 theoretical adults and 20 theoretical children whose assigned values are listed in Supplemental Tables 1 and 2, respectively. RID Equation *1* was used to predict TBS as described in Methods. RID, retinol isotope dilution; TBS, total body stores.

SUPPLEMENTAL FIGURE 4

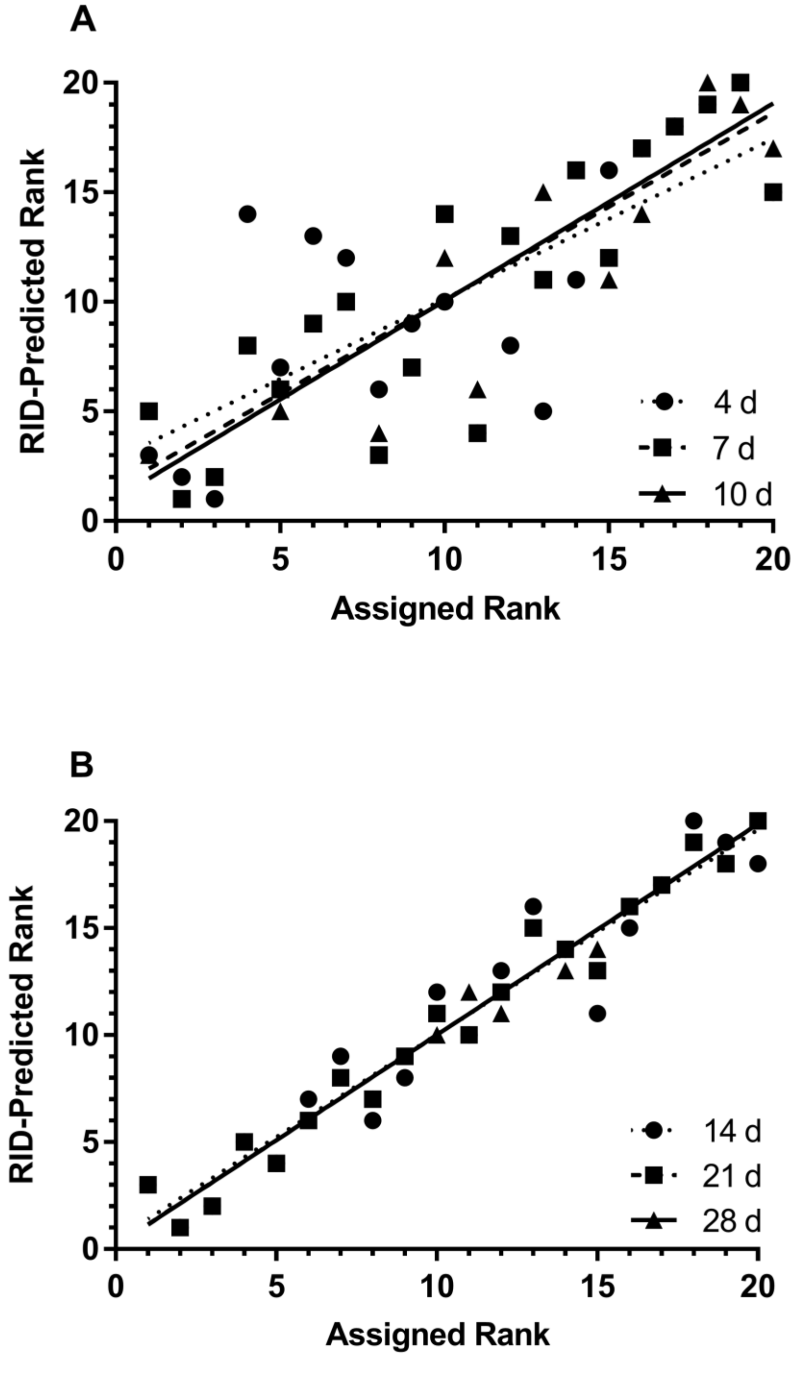

SUPPLEMENTAL FIGURE 4 TBS rank (lowest to highest) for assigned versus RID-predicted TBS for 20 theoretical adults (Supplemental Table 1) at 4, 7, and 10 days (A) and at 14, 21, and 28 days (B). RID Equation *1* (see Methods) was used to predict TBS. Regression equations

are: y = 0.731x + 2.83 (R^2 = 0.534, P = 0.0003) at d 4; y = 0.854x + 1.53 (R^2 = 0.730, P < 0.0001) at d 7; y = 0.901x + 1.04 (R^2 = 0.811, P < 0.0001) at d 10; y = 0.956x + 0.458 (R^2 = 0.915, P < 0.0001) at d 14; y = 0.984x + 0.174 (R^2 = 0.967, P < 0.0001) at d 21; and y = 0.985x + 0.158 (R^2 = 0.970, P < 0.0001) at d 28. RID, retinol-isotope dilution; TBS, total body stores.

SUPPLEMENTAL FIGURE 5

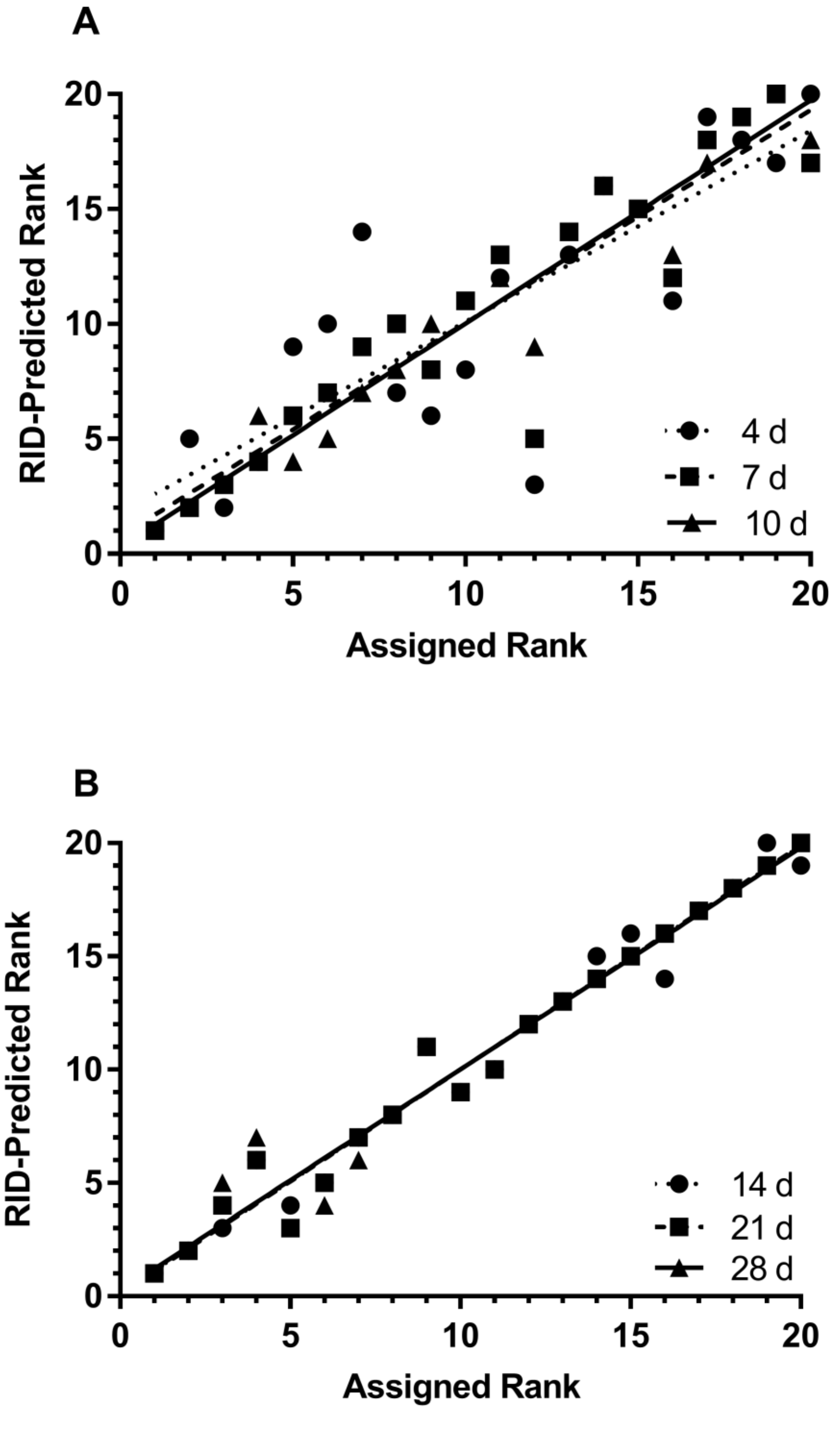

SUPPLEMENTAL FIGURE 5 TBS rank (lowest to highest) for assigned versus RID-predicted TBS for 20 theoretical children (Supplemental Table 2) at 4, 7, and 10 days (A) and at 14, 21, and 28 days (B). RID Equation *1* (see Methods) was used to predict TBS. Regression

equations are: y = 0.832x + 1.77 (R^2 = 0.692, P < 0.0001) at d 4; y = 0.926x + 0.774 (R^2 = 0.858, P < 0.0001) at d 7; y = 0.971x + 0.300 (R^2 = 0.944, P < 0.0001) at d 10; y = 0.985x + 0.158 (R^2 = 0.970, P < 0.0001) at d 14; y = 0.988x + 0.126 (R^2 = 0.976, P < 0.0001) at d 21; and y = 0.979x + 0.221 (R^2 = 0.958, P < 0.0001) at d 28. RID, retinol-isotope dilution; TBS, total body stores.

SUPPLEMENTAL WinSAAM DECK

```
A SAAM31                  S6 SS With 8[05-DEC-2020]
CC MODEL ABS EFF 71.923%
CC MODEL M(6) 682.50
CC MODEL M(7) 10.029
CC MODEL U(1) 1.894936
H PAR
CC INITIAL CONDITION IN COMPARTMENT 1: 100% OF DOSE ADMINISTERED
   IC(1)     1
   L(2,1)    30
   L(0,1)    1.171129E+01  1.181817E+00   7.363635E+01
   L(3,2)=L(2,1)
   DT(3)     2.785130E-02  1.933333E-03   6.239999E-02
   DN(3)    8
   L(4,3)    1
   L(5,4)    1.405752E+000
   L(7,5)    4.553000E+00
   L(5,7)    2.269956E+00
   L(6,5)    2.999945E+00
   L(5,6)    0.02097926
   L(9,6)    0.00099846
   L(8,5)    0.136289391
   DT(8)    0.052
   DN(8)    8
   L(11,8)  1
CC  H STE TO SET UP STEADY STATE SOLUTION
H STE
   M(5)      5.0
   U(1)      1.894936E+00 0.0           100
H DAT
C Insert data values here
CC  PLASMA  TIME (d)  FRACTION OF DOSE   WEIGHT FOR REGRESSION
105                                      FSD=0.05
            0                  0
            0.02083       0
            0.04166       0.000407551
            0.06249       0.00439999
            0.08332       0.0125778
            0.10415       0.023188
            0.12498       0.0345428
            0.14581       0.0455341
            0.16664       0.0555724
            0.18747       0.0644086
            0.2083        0.0720062
            0.22913       0.0784358
            0.24996       0.0838157
            0.27079       0.0882784
            0.29162       0.0919529
            0.31245       0.0949566
            0.33328       0.0973929
```

```
           1                0.0916611
           2                0.0544652
           4                0.0165613
           6                0.00707188
           8                0.00500367
           10              0.00455852
           14              0.00441863
           21              0.00435761
           28              0.00430082
           35              0.00424473
           42              0.00418931
           49               0.00413468
           56               0.00408071
CC CALCULATION OF ABSORPTRION EFFICIENCY
111G(11)
XG(11)=(L(2,1)/(L(2,1)+L(0,1)))*100             1
100
   M(5)
   M(6)
   M(7)
   U(1)
   G(11)
CC SAp
125G(25)
XG(25)=F(5)/M(5)
           1
2          1                            29
CC FaS
135G(35)
XG(35)=(F(6)+F(7))*((F(5)/M(5))/((F(6)+F(7))/
       (M(6)+M(7))))
           1
2          1                            29
CC  Fa
136G(36)
XG(36)=(F(6)+F(7))
           1
2          1                            29
CC  S
137G(37)
XG(37)=((F(5)/M(5))/((F(6)+F(7))/
       (M(6)+M(7))))
           1
2          1                            29
```
